# Unburned Tobacco Smoke Affects Neuroinflammation-Related Pathways in the Rat Mesolimbic System

**DOI:** 10.3390/ijms25105259

**Published:** 2024-05-11

**Authors:** Camilla Morosini, Fabio Vivarelli, Laura Rullo, Emilia Volino, Loredana Maria Losapio, Moreno Paolini, Patrizia Romualdi, Donatella Canistro, Sanzio Candeletti

**Affiliations:** 1 Department of Pharmacy and Biotechnology, Alma Mater Studiorum–University of Bologna, 40126 Bologna, Italy; camilla.morosini2@unibo.it (C.M.); fabio.vivarelli3@unibo.it (F.V.); emilia.volino@studio.unibo.it (E.V.); loredana.losapio2@unibo.it (L.M.L.); moreno.paolini@unibo.it (M.P.); donatella.canistro@unibo.it (D.C.); sanzio.candeletti@unibo.it (S.C.); 2 Department of Biomedical and Neuromotor Sciences, Alma Mater Studiorum–University of Bologna, 40126 Bologna, Italy

**Keywords:** neuroinflammation, heat-not-burn devices, tobacco smoke, VTA, NAc

## Abstract

Tobacco use disorder represents a significant public health challenge due to its association with various diseases. Despite awareness efforts, smoking rates remain high, partly due to ineffective cessation methods and the spread of new electronic devices. This study investigated the impact of prolonged nicotine exposure via a heat-not-burn (HnB) device on selected genes and signaling proteins involved in inflammatory processes in the rat ventral tegmental area (VTA) and nucleus accumbens (NAc), two brain regions associated with addiction to different drugs, including nicotine. The results showed a reduction in mRNA levels for *PPARα* and *PPARγ*, two nuclear receptors and anti-inflammatory transcription factors, along with the dysregulation of gene expression of the epigenetic modulator *KDM6s*, in both investigated brain areas. Moreover, decreased *PTEN* mRNA levels and higher AKT phosphorylation were detected in the VTA of HnB-exposed rats with respect to their control counterparts. Finally, significant alterations in ERK 1/2 phosphorylation were observed in both mesolimbic areas, with VTA decrease and NAc increase, respectively. Overall, the results suggest that HnB aerosol exposure disrupts intracellular pathways potentially involved in the development and maintenance of the neuroinflammatory state. Moreover, these data highlight that, similar to conventional cigarettes, HnB devices use affects specific signaling pathways shaping neuroinflammatory process in the VTA and NAc, thus triggering mechanisms that are currently considered as potentially relevant for the development of addictive behavior.

## 1. Introduction

Tobacco use disorder, commonly known as nicotine dependence, represents the most widespread substance use disorder and is currently one of the greatest public health challenges as it significantly contributes to the development of neoplastic, cardiovascular, and respiratory diseases [1]. The addictive substance in tobacco is nicotine, a natural alkaloid found in the tobacco plant. Inhaled cigarette smoke carries nicotine into the lungs where it is quickly absorbed. After progressing to the systemic arterial circulation, it crosses the blood–brain barrier and diffuses into the brain, where it binds to nicotinic acetylcholine receptors (nAChR), causing the release of neurotransmitters into the bloodstream [2]. In particular, in the central nervous system (CNS), nicotine stimulates the cortical and limbic brain structures, which mediate rewarding motivational effects, including the ventral tegmental area (VTA) and the nucleus accumbens (NAc) [3]. Despite the efforts of health authorities to raise awareness among the population about the risks of smoking, the number of smokers remains high, also due to the negative outcomes of pharmacological and non-pharmacological approaches toward smoking cessation proposed to date [4]. Heat-not-burn (HnB) systems, the latest devices in the electronic cigarette family, are advertised as low-risk devices for smoking cessation support. This brand-new system heats rather than burns tobacco to create mainstream smoke that the user inhales. Despite these products having been promoted as a substitute for traditional cigarette smoking, until now, this solution has not proven to be optimal; HnB devices may increase the risk of dual or poly-tobacco product use among young adults, including current exclusive e-cigarette users [5,6,7]. Despite the amount of nicotine in HnB devices being quite similar to the mean nicotine quantity reported for conventional combustible cigarettes, smokers who try to switch to HnB devices to quit smoking have reported experiencing withdrawal symptoms from traditional cigarettes and cravings for them, often leading to dual use [8,9]. In addition, nicotine dependence levels, measured using the Fagerstrom Test in young adults, have been reported to be significantly higher among users of exclusive electronic devices than in traditional tobacco smokers [10].

Furthermore, it is important to emphasize that the effects induced by nicotine exposure through these devices are still poorly understood, and recent studies seem to confirm the development of oxidative and neuroinflammatory phenomena associated with the use of these systems, which could significantly affect the proper functioning of the CNS. In fact, our previous work, along with other literature results, has shown how exposure to HnB devices can induce neuroinflammation, promoting an increase in specific cytokines and the dysregulation of some transcriptional activity regulators including peroxisome proliferator-activated receptors (PPARs) [11,12].

A considerable amount of evidence has highlighted the relevant role of PPARs in the control of different inflammatory responses. In particular, this function is related to the PPARα and PPARγ isoform abilities to transrepress the activities of many activated transcription factors, including nuclear factor kappa-light-chain-enhancer of activated B cells (NF-κB), signal transducers and activators of transcription (STATs), and activator protein 1 (AP1) [13].

In addition to ligand binding, the genomic activity of PPARs is modulated by the phosphorylation of a serine residue by MAPKs, such as extracellular signal-regulated protein kinases-1/2 (ERK-1/2) or by their nucleocytoplasmic compartmentalization through ERK activation [14]. Thus, emerging evidence linking dysfunctional ERK 1/2 signaling to inflammatory diseases [15] has been recently reported. In turn, PPARs promote the gene expression of “phosphatase and tensin homolog deleted on chromosome 10” (PTEN), known to negatively regulate the PI3K/AKT (phosphatidylinositol 3-kinase/protein kinase B) pathways [16], thus leading to anti-inflammatory/neuroprotective effects. 

Furthermore, a crucial role of epigenetic enzymes, such as the histone demethylases KDM6s, in the regulation of PPARs has been reported [17].

On these bases and given the widespread use of HnB devices, we sought to better analyze the role of neuroinflammatory processes in the development of nicotine use disorder [18]. Hence, in light of our previous results [11] in the prefrontal cortex (PFC), we focused this investigation on two other relevant nodes of the mesocorticolimbic reward circuitry represented by VTA and NAc. To this end, we evaluated the effects of prolonged exposure to HnB mainstream smoke on the *PPARs*, *PTEN*, and *KDM6s* gene expression as well as on ERK1/2 and AKT phosphorylated protein levels.

## 2. Results

### 2.1. Gene Expression Analysis

**Figure 1:**
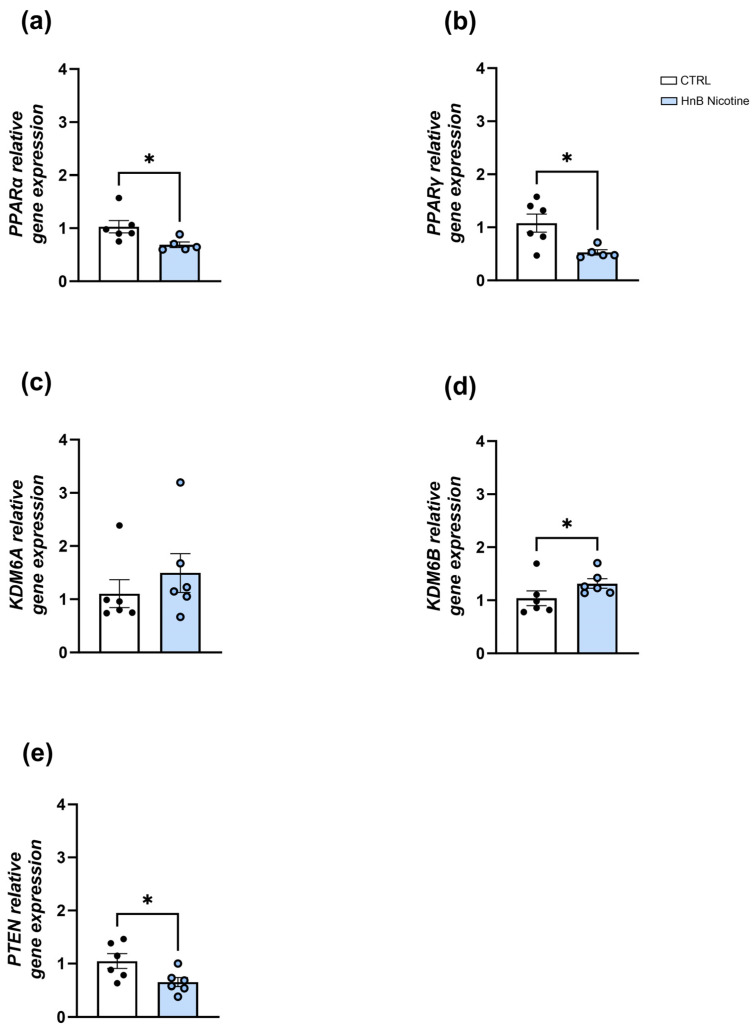
Effects of the nicotine exposure via an HnB device on the relative gene expression of *PPARα* (**a**), *PPARγ* (**b**), *KDM6A* (**c**), *KDM6B* (**d**), and *PTEN* (**e**) in the rat VTA. Data represent 2^−ΔΔCT^ values calculated using the ΔΔCT method and are expressed as the means ± SEM; * *p* < 0.05; two-tailed *t*-test.

In the VTA, downregulation of the mRNA levels of the nuclear receptors PPARα (0.69 ± 0.05 vs. 1.03 ± 0.12, *p* < 0.05; Figure 1a) and PPARγ (0.53 ± 0.05 vs. 1.08 ± 0.17, *p* < 0.05; Figure 1b) was observed in the smoking group. No significant alterations in the epigenetic enzyme histone demethylase *KDM6A* gene expression (1.49 ± 0.36 vs. 1.10 ± 0.26, *p* > 0.05, Figure 1c) were detected in the treated group; however, data analysis revealed a significant increase in the gene expression of the isoform *KDM6B* (1.31 ± 0.09 vs. 1.03 ± 0.15, *p* < 0.05; Figure 1d). The unpaired *t*-test showed downregulation of *PTEN* mRNA levels (0.65 ± 0.09 vs. 1.05 ± 0.14, *p* < 0.05; Figure 1e) in the animals exposed to nicotine via an HnB device compared to those in the control group. 

**Figure 2:**
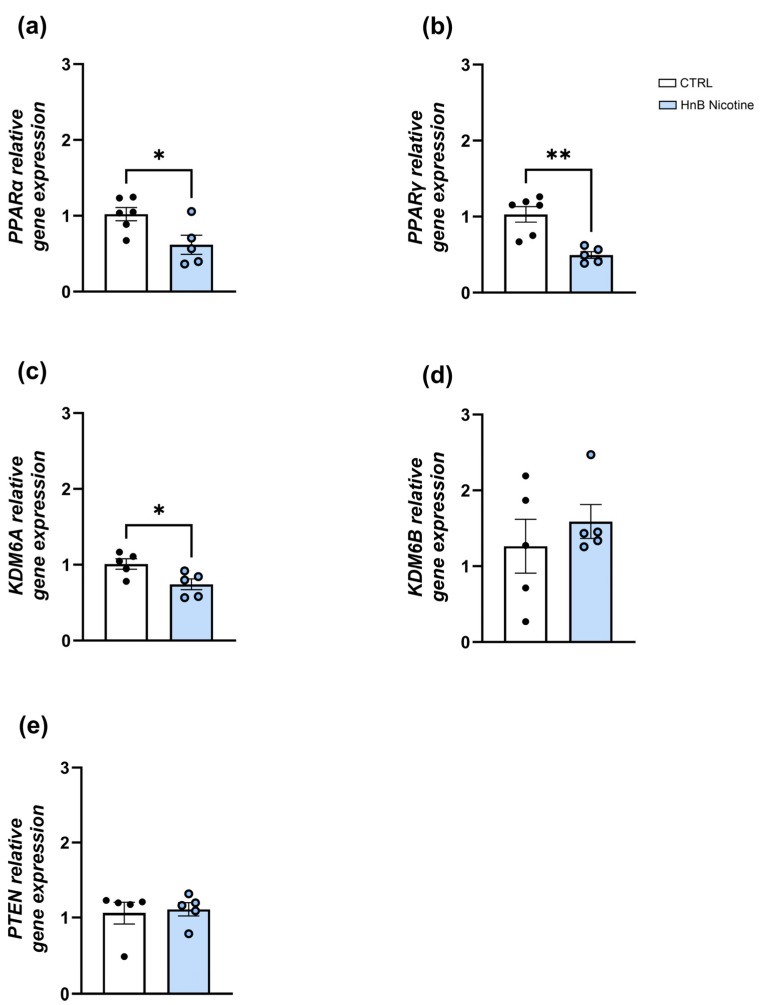
Effects of the nicotine exposure via an HnB device on the relative gene expression of *PPARα* (**a**), *PPARγ* (**b**), *KDM6A* (**c**), *KDM6B* (**d**), and *PTEN* (**e**) in the rat NAc. Data represent 2^−ΔΔCT^ values calculated using the ΔΔCT method and are expressed as the means ± SEM; * *p* < 0.05; ** *p* < 0.01, two-tailed *t*-test.

Similarly, in the NAc, lower levels of nuclear receptors *PPARα* (0.62 ± 0.13 vs. 1.02 ± 0.09, *p* < 0.05; Figure 2a) and *PPARγ* (0.49 ± 0.04 vs. 1.03 ± 0.12, *p* < 0.01; Figure 2b) gene expression were observed in the group exposed to nicotine via an HnB device compared to those in the control group. The unpaired *t*-test revealed downregulation of *KDM6A* (0.74 ± 0.07 vs. 1.01 ± 0.07, *p* < 0.05, Figure 2c) with no significant change in the *KDM6B* mRNA expression (1.59 ± 0.22 vs. 1.26 ± 0.35, *p* > 0.05; Figure 2d) in the smoking group. Moreover, no significant changes in *PTEN* (1.11 ± 0.09 vs. 1.06 ± 0.15, *p* > 0.05; Figure 2e) gene expression were detected in the treated group.

### 2.2. Protein Analysis

**Figure 3:**
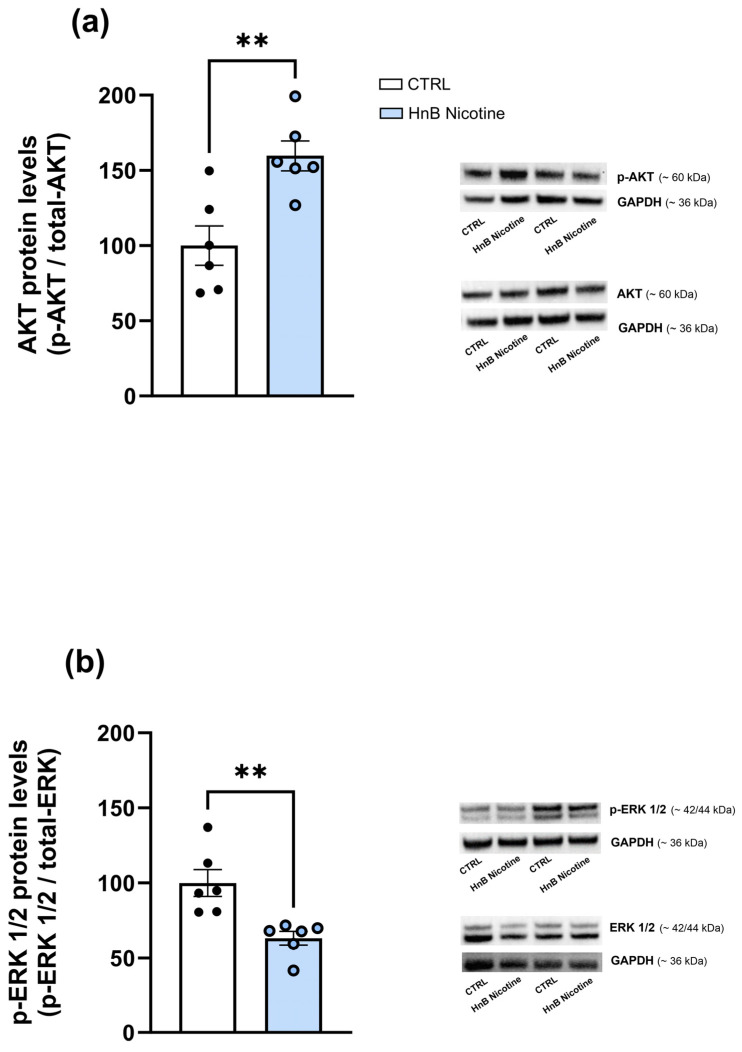
Effects of nicotine exposure via an HnB device on AKT (~60 kDa) (**a**) and ERK 1/2 (~42/44 kDa) (**b**) phosphorylated protein levels in the rat VTA. A representative blot is reported alongside the histogram. Bars represent the means ± SEM; ** *p* < 0.01; two-tailed *t*-test.

**Figure 4:**
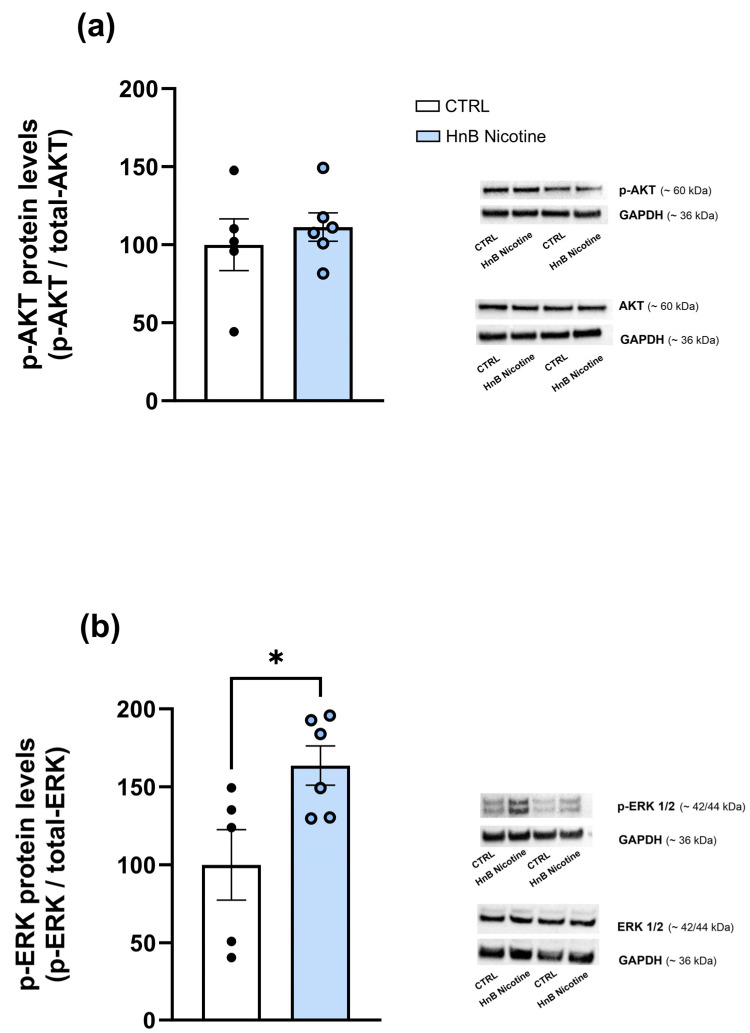
Effects of nicotine exposure via an HnB device on AKT (~60 kDa) (**a**) and ERK 1/2 (~42/44) (**b**) phosphorylated protein levels in the rat NAc. A representative blot is reported alongside the histogram. Bars represent the means ± SEM; * *p* < 0.05; two-tailed *t*-test.

In the VTA, data analysis showed higher levels of phosphorylated AKT (159.71 ± 9.91 vs. 100 ± 13, *p* < 0.01; Figure 3a) protein along with a decrease in the phosphorylation of ERK (63.12 ± 4.62 vs. 100 ± 8.87, *p* < 0.01; Figure 3b) in the smoking group compared the control one. However, no alterations were observed in the phosphorylation of AKT (111.32 ± 9.12 vs. 100 ± 16.58, *p* > 0.05; Figure 4a) in the NAc of rats exposed to nicotine, although increased levels of phosphorylated ERK (163.65 ± 12.57 vs. 100 ± 22.57, *p* < 0.05; Figure 4b) were observed compared to the control group animals. 

## 3. Discussion

The present study investigated the effects of prolonged nicotine exposure (delivered by an HnB device) on the transcriptomic and epigenetic modulators PPARs and KDMs and the possible involvement of ERK1/2 and PTEN-AKT cellular cascade in the neuroadaptive changes underlying nicotine addiction. In particular, our results suggest that prolonged exposure to HnB mainstream smoke is able to differently alter these signaling pathways in VTA and NAc and highlight that this dysregulation might participate in some neuroinflammatory processes associated with the development of tobacco use disorder. 

Interestingly, the data reported herein show that nicotine, similarly to other drugs of abuse (e.g., cocaine and alcohol) significantly alters the expression of inflammation regulatory genes in the brain regions chiefly involved in reward processes [19,20]. In particular, a significant increase in *KDM6B* mRNA levels, along with downregulation of both *PPARα* and *PPARγ* gene expression, has been detected in the VTA of nicotine-exposed rats. Consistent with previous studies showing that KDM enzymes inhibit the expression and activity of the nuclear receptors PPARs [17,21], these findings support the hypothesis that the inflammatory process triggered by nicotine inhaled via HnB mainstream smoke [22] could be epigenetically influenced by KDMs and could entail PPAR regulation. Although both KDM6A and KDM6B have been reported to promote inflammatory processes through the removal of trimethyl marks at lysine 27 residues of H3 in gene promoter regions, our data seem to suggest the major involvement of the KDM6B isoform in modulating the inflammatory response within this specific brain region. Notably, other studies also suggest the contribution of KDM6B to the increase in pro-inflammatory cytokines (e.g., IL-6 and IL-1β) caused by different drugs of abuse [19,20]. 

Different from what has been previously observed in the PFC [11], no significant alterations in *KDM6A* gene expression have been detected in the VTA, thus suggesting reduced involvement of this specific epigenetic enzyme isoform in the alterations induced by prolonged nicotine exposure in this area.

Likewise, the reduction in *PPARα* and *PPARγ* mRNA levels could contribute to the VTA inflammatory response during the development of HnB tobacco-induced dependence. The role of PPARs in substance use disorder (SUD) has been widely proposed [23] and PPAR agonists (e.g., pioglitazone and rosiglitazone) have been found to be highly effective in reducing drug-seeking behavior, including nicotine cravings [24,25,26]. Indeed, these nuclear receptors, besides regulating the expression of multiple genes associated with GABAergic and glutamatergic transmission in several mesocorticolimbic brain regions [27], are known to modulate inflammatory responses by inhibiting pro-inflammatory signaling pathways, such as NF-κB activation [28]. 

Interestingly, our results indicate a reduction in *PTEN* mRNA levels in the VTA of HnB-exposed rats. In light of PTEN’s relationship with PPARs and its role in suppressing the PI3K/AKT signaling pathway [29,30], the increased AKT phosphorylation observed herein highlights a link between tobacco smoke and the inflammatory state [16]. 

Notably, PTEN loss disrupts normal connectivity within neuronal circuits by affecting the balance between excitation and inhibition [31,32]. In addition, chronic nicotine exposure has been observed to enhance excitatory glutamate neurotransmission and decrease inhibitory GABA neurotransmission, probably through the desensitization of nACh receptors [33]. Thus, given PTEN’s function in brain and synaptic connectivity [32], it is conceivable that prolonged nicotine inhalation might contribute to an increase in excitatory synaptic connectivity at the VTA level, thus leading to the development of pathological dependence. 

A reduction in ERK phosphorylation has been detected in this brain region. Although controversial results exist about the involvement of ERK in inflammation [34,35], a role for the dysregulation of this kinase has been proposed in addiction development [36]. Similarly to what was observed in the VTA, HnB mainstream smoke reduced *PPAR* mRNA levels in the NAc. However, no significant changes in *KDM6B* and a reduction in *KDM6A* gene expression were observed, thus suggesting the involvement of other epigenetic mechanisms in PPAR and inflammatory process regulation in this brain area. Indeed, alterations in DNA methylation as well as alterations in histone acetylation have been reported after prolonged exposure to nicotine in different areas of the reward circuitry [37] and seem to concur differently in the disruption of the cell signaling pathways involved in nicotine dependence. Additionally, exposure of rats to HnB tobacco smoke did not induce alterations in the PTEN-AKT signaling cascade in the NAc.

Despite the lack of alterations in some of the investigated targets, HnB mainstream smoke seems capable of inducing neurochemical changes in the NAc anyway. In fact, the increase in ERK phosphorylation herein observed highlights the ability of these devices to promote the disruption of intracellular pathways associated with the development and maintenance of a neuroinflammatory state. In this regard, several studies have demonstrated that ERK signaling is critical for the regulation of the pro-inflammatory response [38,39] as well as the regulation of PPAR activation [34]. 

Indeed, ERK activation can either modulate the genomic activity of these anti-inflammatory transcription factors through the phosphorylation of a serine residue or regulate their nuclear/cytoplasm localization [14]. 

Moreover, nicotine’s ability to elevate ERK phosphorylation in the rat NAc has already been shown [40,41], thus supporting the role of this kinase in neuronal plasticity induced by nicotine dependence [42] regardless of the smoking device used. 

Overall, the alterations observed herein seem to suggest that the prolonged HnB tobacco exposure-induced disruption of the PTEN-AKT signaling pathway in the VTA could enhance dopamine release in the NAc, probably inducing alterations in excitatory synaptic connectivity. In turn, this increase in dopaminergic firing could trigger ERK phosphorylation in the NAc, through the activation of dopamine receptor signaling, thus participating in the development of nicotine addictive behavior [36].

In light of these results and together with the previous observations in the prefrontal cortex [11], it seems that the molecular pathways involved in inflammatory process regulation are engaged differently along the starting or projection nodes of the mesocorticolimbic pathway.

In summary, these findings offer new insights into cellular pathways involved in the inflammatory processes associated with nicotine use disorder. A potential limitation of this study may be represented by the employment of male rats only; therefore, we cannot extend our findings to female subjects. Although further studies are required to gain a deeper understanding of the long-term effects of HnB devices on brain functions, the results presented herein indicate that HnB devices can affect specific signaling pathways, shaping neuroinflammatory processes likely relevant in the development of addictive behavior, in the rat VTA and NAc. 

## 4. Materials and Methods

### 4.1. Animal Model and Nicotine Exposure 

The animal experiments were planned according to EU Directive (2010/63/EU) guidelines, and the protocol was approved by the Committee on Animal Research and Ethics of the University of Bologna and by the Italian Ministry of Health (permit number 360/216-PR; 2683215). Twelve male 7-week-old Sprague Dawley rats (ENVIGO RMS SRL, San Pietro al Natisone, Italy) were housed under standard conditions (12 h light–dark cycle, 22 °C, 60% humidity), with free access to water and food. After one week of acclimatization, the animals were separated into two groups, a control (n = 6 rats) and an exposed (n = 6 rats) group. The rats assigned to the latter were transferred to an inhalation chamber (two animals per chamber), in which they were exposed using whole-body mode to nicotine (delivered by the heat-not-burn THS 2.2 model produced by PMI) for a total period of 28 days (5 days/week) as previously described [11,43,44]. Total mainstream smoke exposure was limited to 8 tobacco sticks/day/chamber, never exceeding 3h/day. The control group was not exposed to any treatment, and the animals spent the same time in the inhalation chamber as the treated ones. The Committee on Animal Research and Ethics monitored the animals throughout the entire experimental program. The animal studies were performed in agreement with ARRIVE guidelines [45]. 

At the end of treatment, the rats were sacrificed, and the VTA and NAc were dissected from each rat according to the Rat Brain Atlas [46] and stored at −80 °C for successive molecular analysis.

### 4.2. RNA Extraction and Gene Expression Analysis Using Real-Time qPCR

Total RNA was extracted according to the method of Chomczynski and Sacchi [47]. Each sample was subjected to DNAse treatment and converted to cDNA as previously described [48,49]. The relative abundance of each mRNA of interest was assessed via real-time qRT-PCR using the Sybr Green gene expression Master Mix (Life Technologies, Carlsbad, CA, USA) in a Step One Real-Time PCR System (Life Technologies). The relative expression of different gene transcripts was calculated using the delta–delta Ct (DDCt) method and converted to the relative expression ratio (2^−DDCt^) for statistical analysis. All data were normalized to the housekeeping gene glyceraldehyde-3-phosphate dehydrogenase (*GAPDH*). The specificity of each PCR product was determined via melting curve analysis, constructed in the range of 60 °C to 95 °C. The primers used for PCR amplification were designed using Primer 3, and their sequences are reported as follows:

− *GAPDH* Forward 5′-AGACAGCCGCATCTTCTTGT-3′;
  Reverse 5′-CTTGCCGTGGGTAGAGTCAT-3′;
− *KDM6A* Forward 5′-TTTGGTCTACTTCCATTACAATGCA-3′;
  Reverse 5′-AAGCCCAAGTCGTAAATGAATTTC-3′;
− *KDM6B* Forward 5′-ACCGCCTGCGTGCCTTAC-3′;
  Reverse 5′-GTGTTGCTGCTGCTGCTACTG-3′;
− *PTEN* Forward 5′-TGGATTCGACTTAGACTTGACCT-3′;
  Reverse 5′-GCGGTGTCATAATGTCTCTCAG-3′;
− *PPAR*α Forward 5′-TGGAGTCCACGCATGTGAAG-3′;
  Reverse 5′-TTGTCGTACGCCAGCTTTAGC-3′;
− *PPAR*γ Forward 5′-CTGTTCGTACGCCAGCTTTAGC-3′;
  Reverse 5′-GCTCATATCTGTCTCCGTCTTCTT-3′;

### 4.3. SDS-Page and Immunoblotting

Protein extraction was performed using N-PER™ Neuronal Protein Extraction Reagent (Thermo Scientific, Waltham, MA, USA) following the manufacturer’s procedures. A Halt protease and phosphate inhibitor cocktail (Thermo Scientific) was added in accordance with datasheet recommendations. Protein quantification was performed using a Pierce BCA protein assay kit (Thermo Scientific). An equal amount of protein (40 μg protein) was separated on Bolt 4–12% Bis-tris Plus gels (Thermo Scientific) and transferred to 0.45 μm PVDF membranes (Thermo Scientific). Blots were blocked for one hour at room temperature with 5% non-fat dry milk in TBS-T (Tris-buffered saline with 0.1% Tween-20) and then incubated overnight at 4 °C with the following primary antibodies: phospho-AKT (1:1000, cod. no. 4060, Cell Signaling, Boston, MA, USA), AKT (1:1000, cod. no. 9272, Cell Signaling), and phospho-ERK1/ERK2 (1:500, cod. no. 700012, Invitrogen, Carlsbad, CA, USA), ERK1/ERK2 (1:500, cod. no. 13-6200, Invitrogen). The results were standardized using glyceraldehyde 3-phosphate dehydrogenase (GAPDH) (1:1000; cod. no. MA5−15738, Invitrogen). The membranes were washed three times with TBS-T and then incubated for 1 h at room temperature with peroxidase-linked anti-rabbit (1:3000, cod. no. 65-6120, Invitrogen) or anti-mouse (1:3000, cod. no. 62-6520, Invitrogen) secondary antibody. Immunocomplexes were visualized via chemiluminescence using a Chemidoc MP Imaging System (Bio-Rad Laboratories, Hercules, CA, USA) and analyzed with the software ImageJ 1.7.0. The gels were each run twice, and the results represent the average from two different Western blots. In Supplementary Figures S1 and S2 results for phospho- and total AKT as well as for phospho- and total ERK, separately detected, are presented. Full-size original immunoblots of protein expression levels are presented in the Supplementary Materials. 

### 4.4. Statistical Analysis 

Data were evaluated using the Shapiro–Wilk test to confirm the normality of the distribution and Grubb’s test to identify outliers. Statistical analysis was performed using the two-tailed unpaired *t*-test or Mann–Whitney test in case of non-normal distribution. All statistical analyses were performed using GraphPad Prism version 10.0.2 for Windows, (GraphPad Software, Boston, Massachusetts USA). The results are expressed as means ± standard error of the mean (SEM) (*n* = 5–6 animals/group). 

## Data Availability

Data supporting the findings of this study are available upon reasonable request.

## Supplementary Materials

The following supporting information can be downloaded at: https://www.mdpi.com/article/10.3390/ijms25105259/s1.
