# Supplementary material for: Unburned Tobacco Smoke Affects Neuroinflammation-Related Pathways in the Rat Mesolimbic System
Source: Int J Mol Sci. 2024 May 11;25(10):5259. doi: 10.3390/ijms25105259 (PMC11120663; doi:10.3390/ijms25105259)
Supplement: Supplementary file 1 [file ijms-25-05259-s001.zip › ijms-2982824-Supplementary Material.pdf]

Supplementary Material

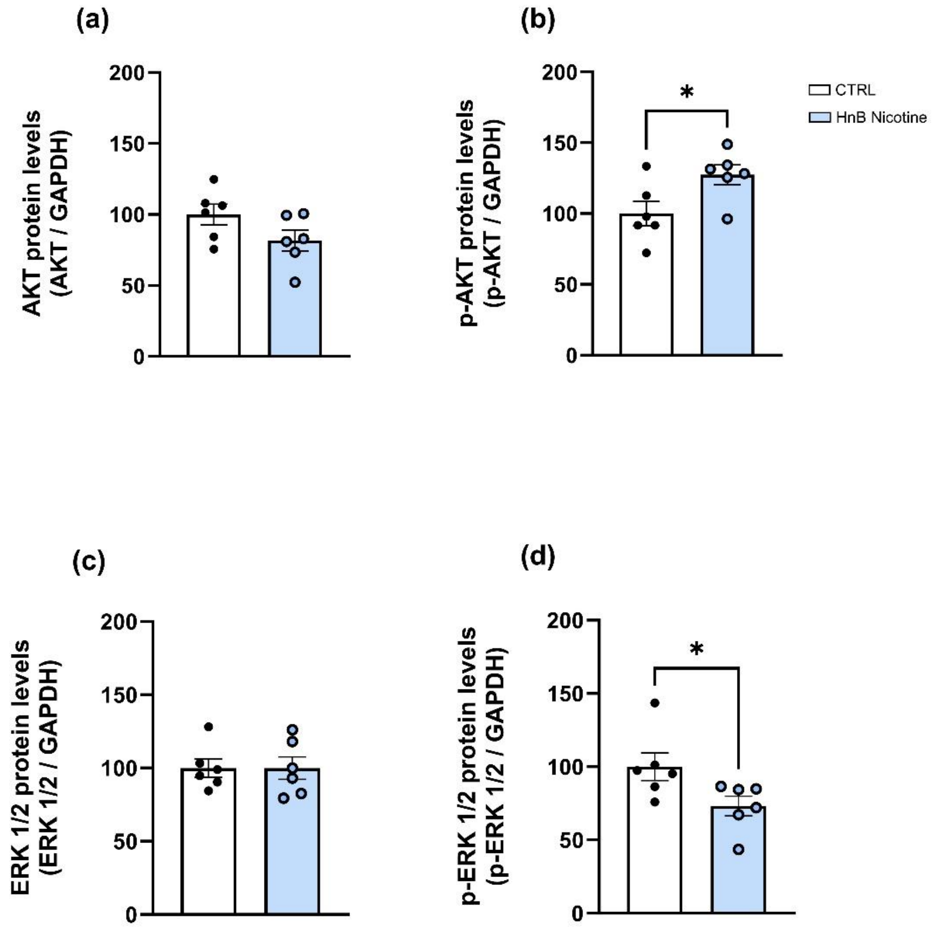

Figure S1. Effects of the nicotine exposure via an HnB device on AKT (a), p-AKT (b), ERK 1/2 (c) and p-ERK 1/2 (d) protein levels in the rat VTA. Bars represent the means ± SEM; * $p < 0.05$; two-tailed t-test.

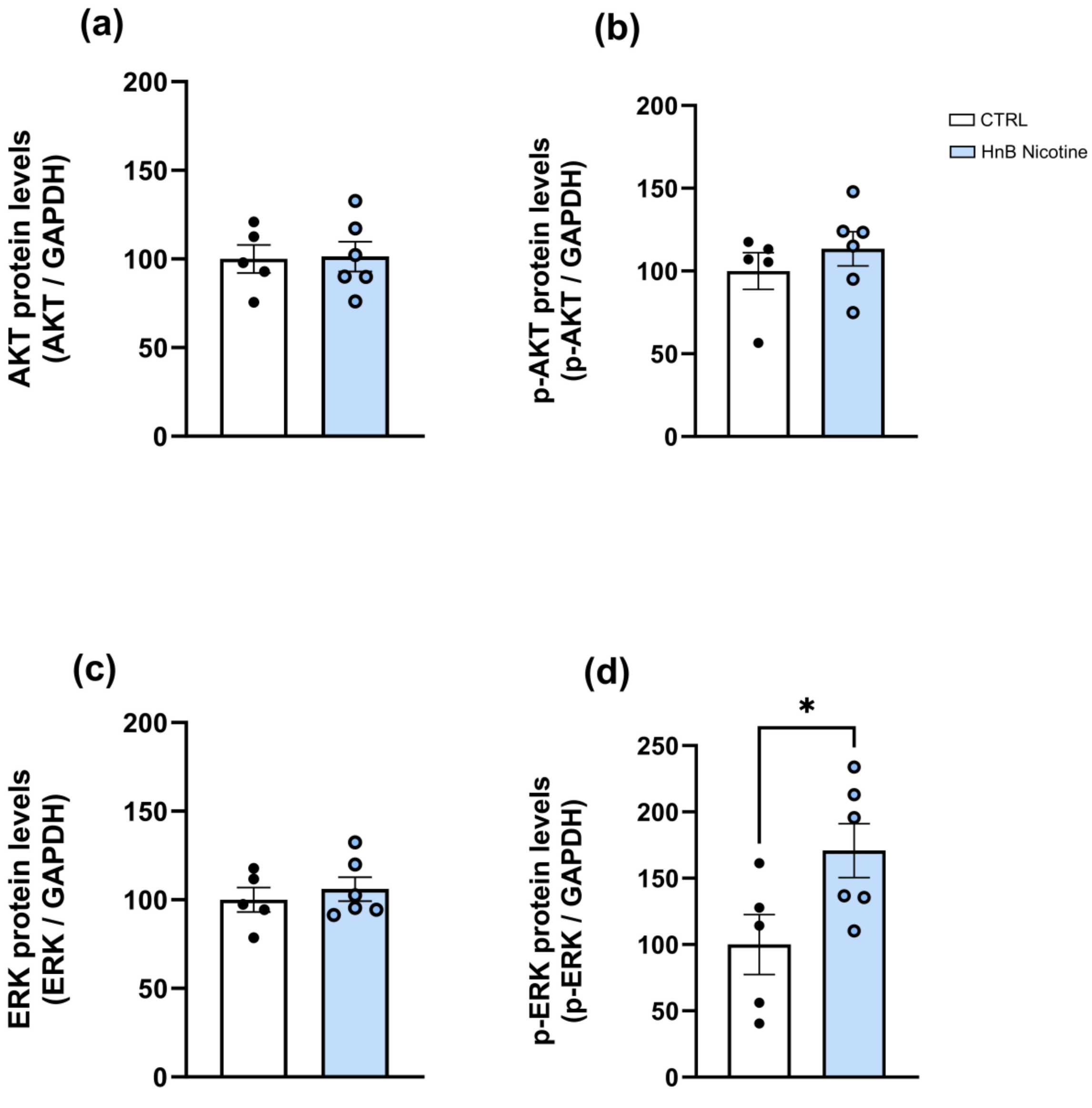

Figure S2. Effects of the nicotine exposure via an HnB device on AKT (a), p-AKT (b), ERK 1/2 (c) and p-ERK 1/2 (d) protein levels in the rat NAc. Bars represent the means ± SEM; * $p < 0.05$; two-tailed t-test.

WESTERN BLOT IMAGES

Nucleus Accumbens

GEL A

p-AKT (~ 60 kDa)

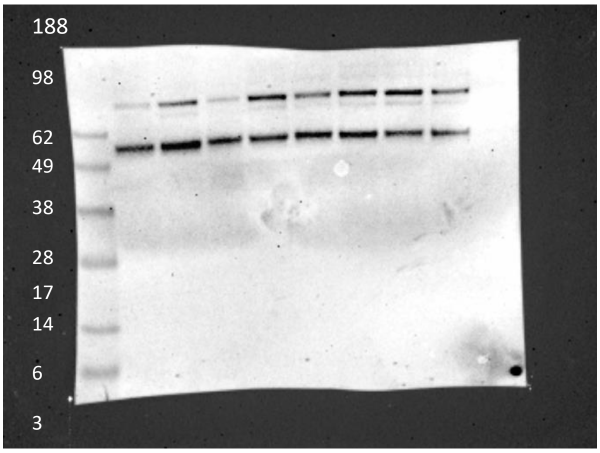

1 CTRL 1 HnB 2 CTRL 2 HnB 3 CTRL 3 HnB 4 CTRL 4 HnB

Representative bands in figure 4a

GAPDH (~ 36 kDa)

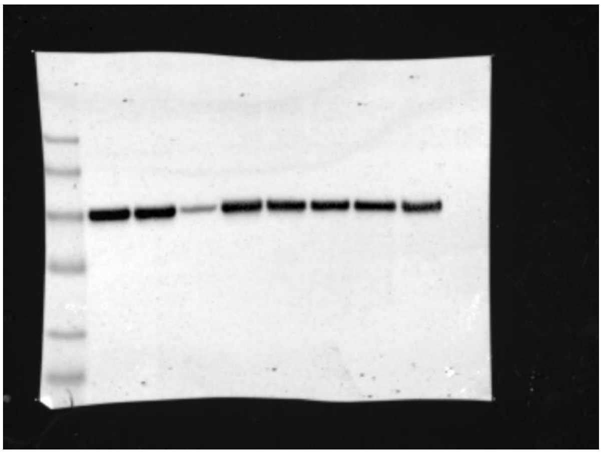

1 CTRL 1 HnB 2 CTRL 2 HnB 3 CTRL 3 HnB 4 CTRL 4 HnB

Representative bands in figure 4a

GEL A

AKT (~ 60 kDa)

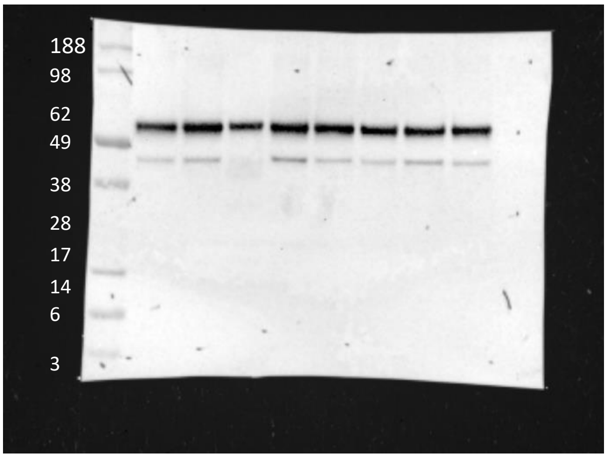

1 CTRL 1 HnB 2 CTRL 2 HnB 3 CTRL 3 HnB 4 CTRL 4 HnB

Representative bands in figure 4a

GAPDH (~ 36 kDa)

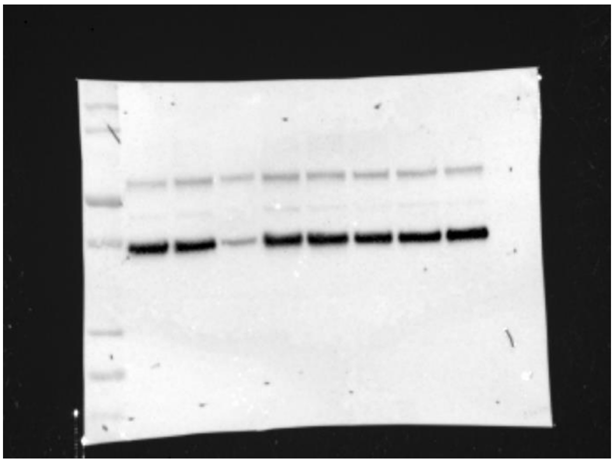

1 CTRL 1 HnB 2 CTRL 2 HnB 3 CTRL 3 HnB 4 CTRL 4 HnB

Representative bands in figure 4a

Nucleus Accumbens

GEL B

p-AKT (~ 60 kDa)

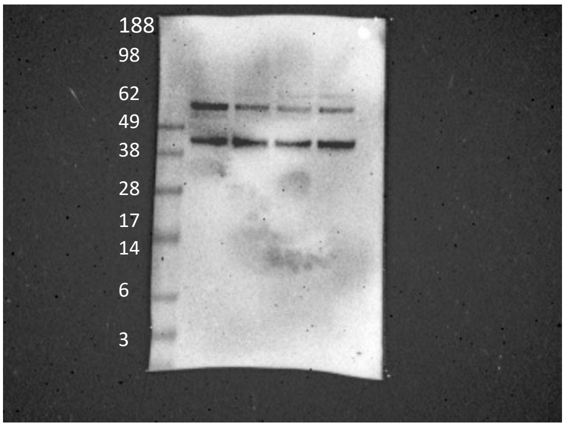

5 CTRL 5 HnB 6 CTRL 6 HnB

GAPDH (~ 36 kDa)

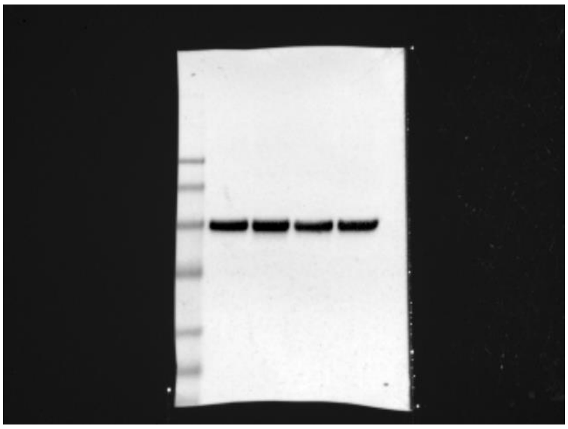

5 CTRL 5 HnB 6 CTRL 6 HnB

GEL B

AKT (~ 60 kDa)

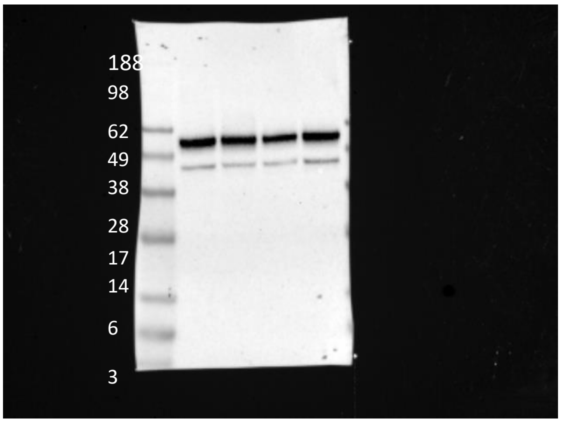

5 CTRL 5 HnB 6 CTRL 6 HnB

GAPDH (~ 36 kDa)

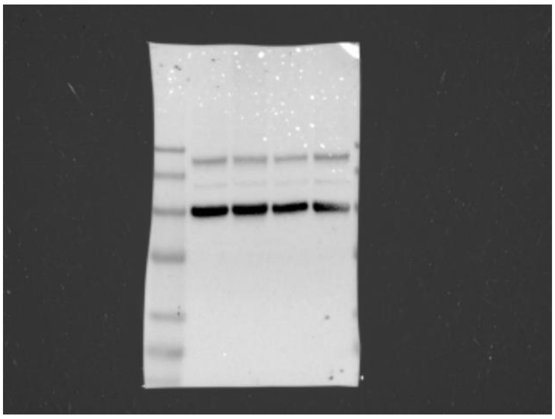

5 CTRL 5 HnB 6 CTRL 6 HnB

Nucleus Accumbens

GEL C

p-AKT (~ 60 kDa)

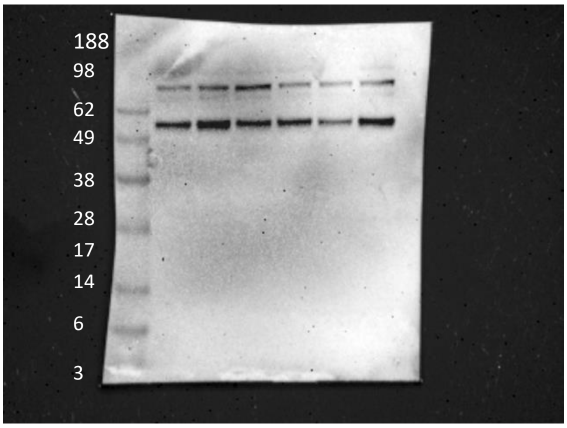

3 CTRL 1 HnB 4 CTRL4 HnB 5 CTRL 6 HnB

GAPDH (~ 36 kDa)

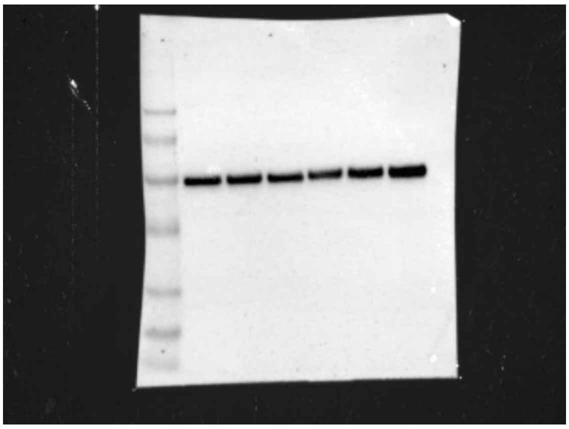

3 CTRL 1 HnB 4 CTRL 4 HnB 5 CTRL 6 HnB

GEL C

AKT (~ 60 kDa)

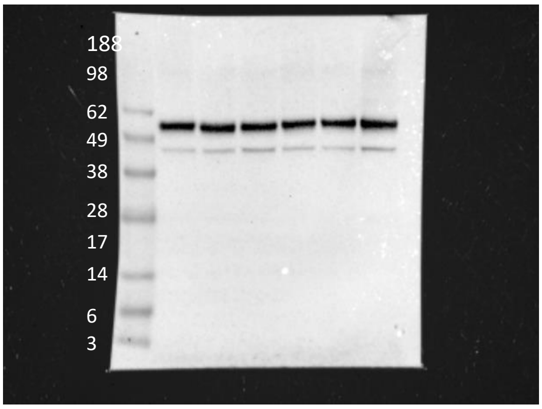

3 CTRL 1 HnB 4 CTRL 4 HnB 5 CTRL 6 HnB

GAPDH (~ 36 kDa)

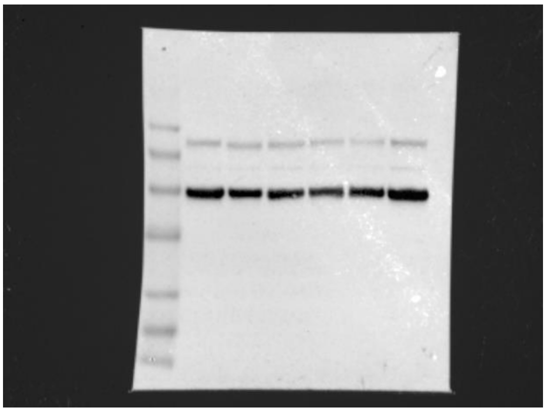

3 CTRL 1 HnB 4 CTRL 4 HnB 5 CTRL 6 HnB

Nucleus Accumbens

GEL D

p-AKT (~ 60 kDa)

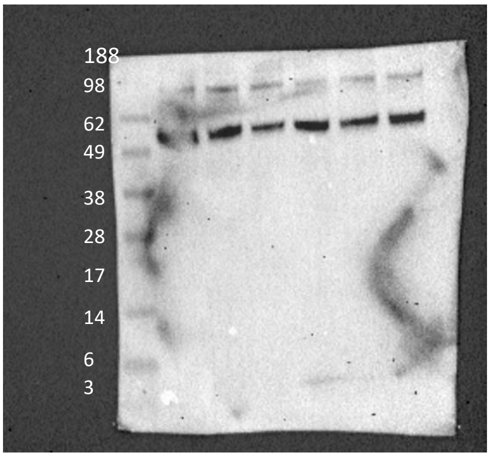

1 CTRL 6 CTRL 2 HnB 3 HnB 4 HnB 5 HnB

GAPDH (~ 36 kDa)

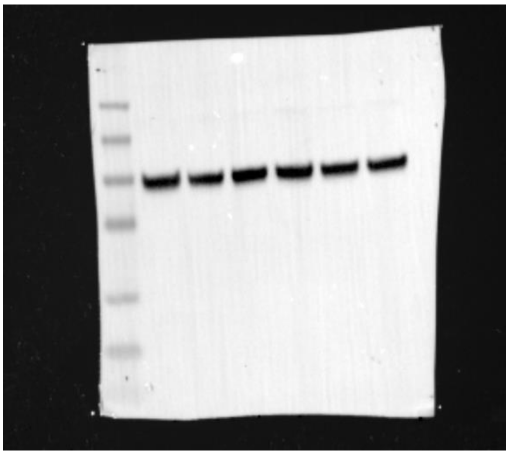

1 CTRL 6 CTRL 2 HnB 3 HnB 4 HnB 5 HnB

GEL D

AKT (~ 60 kDa)

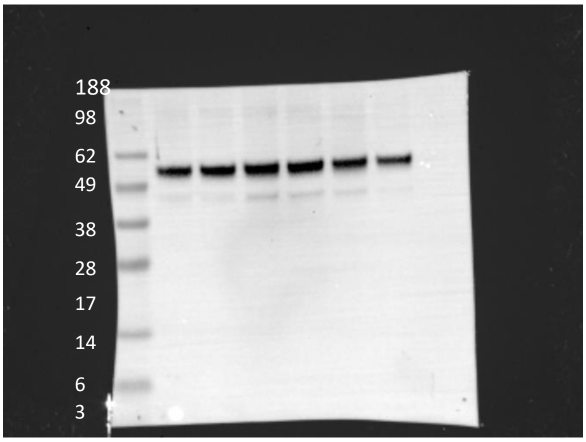

1 CTRL 6 CTRL 2 HnB 3 HnB 4 HnB 5 HnB

GAPDH (~ 36 kDa

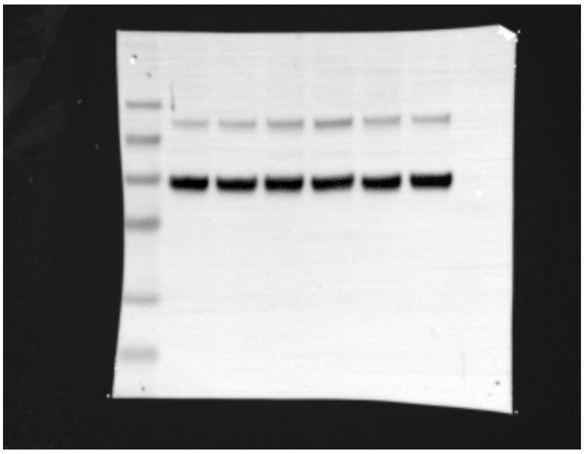

1 CTRL 6 CTRL 2 HnB 3 HnB 4 HnB 5 HnB

Nucleus Accumbens

GEL A

p-ERK 1/2 (~ 42/44 kDa)

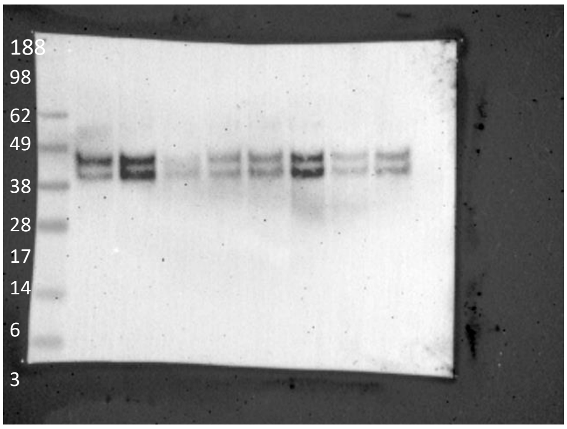

1 CTRL 1 HnB 2 CTRL 2 HnB 3 CTRL 3 HnB 4 CTRL 4 HnB

Representative bands in figure 4b

GAPDH (~ 36 kDa)

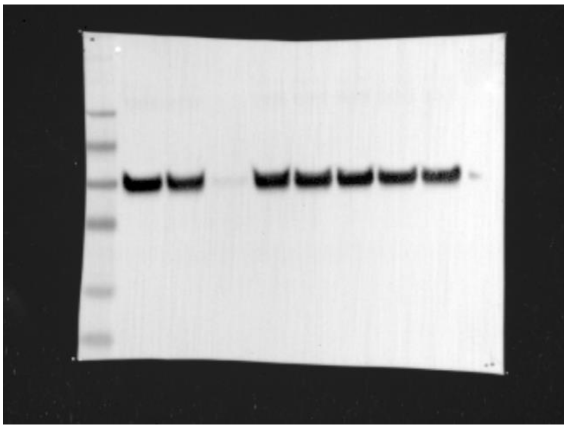

1 CTRL 1 HnB 2 CTRL 2 HnB 3 CTRL 3 HnB 4 CTRL 4 HnB

Representative bands in figure 4b

GEL A

ERK 1/2 (~ 42/44 kDa)

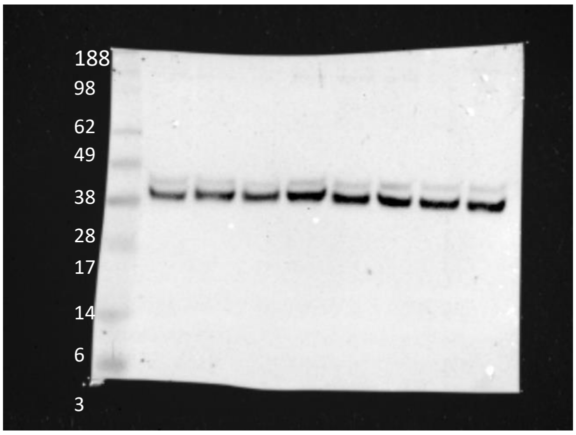

1 CTRL 1 HnB 2 CTRL 2 HnB 3 CTRL 3 HnB 4 CTRL 4 HnB

Representative bands in figure 4b

GAPDH (~ 36 kDa)

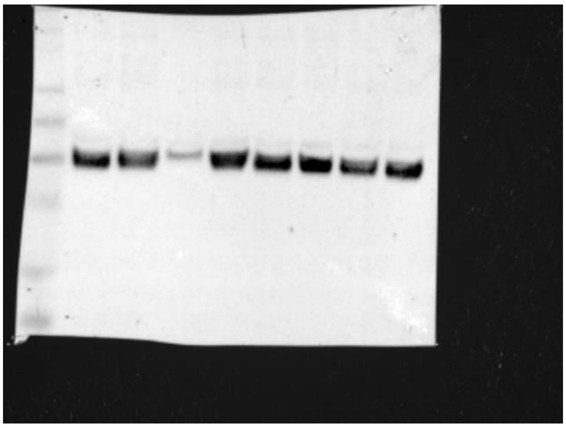

1 CTRL 1 HnB 2 CTRL 2 HnB 3 CTRL 3 HnB 4 CTRL 4 HnB

Representative bands in figure 4b

Nucleus Accumbens

GEL B

p- ERK 1/2 (~ 42/44 kDa)

GAPDH (~ 36 kDa)

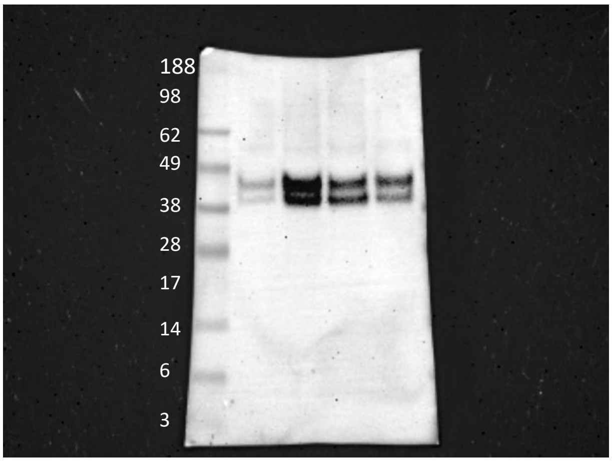

5 CTRL 5 HnB 6 CTRL 6 HnB

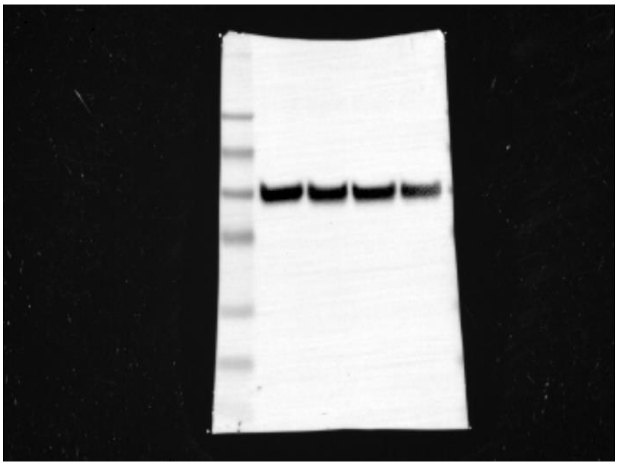

5 CTRL 5 HnB 6 CTRL 6 HnB

GEL B

ERK 1/2 (~ 42/44 kDa)

GAPDH (~ 36 kDa)

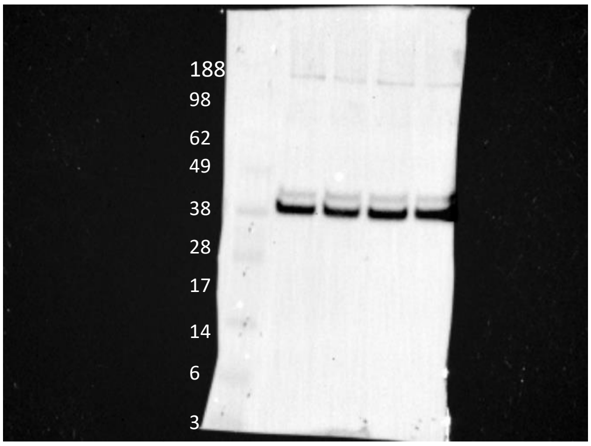

5 CTRL 5 HnB 6 CTRL 6 HnB

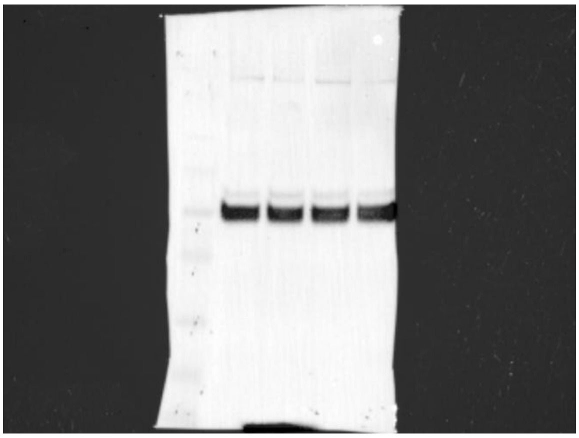

5 CTRL 5 HnB 6 CTRL 6 HnB

Nucleus Accumbens

GEL C

p- ERK 1/2 (~ 42/44 kDa)

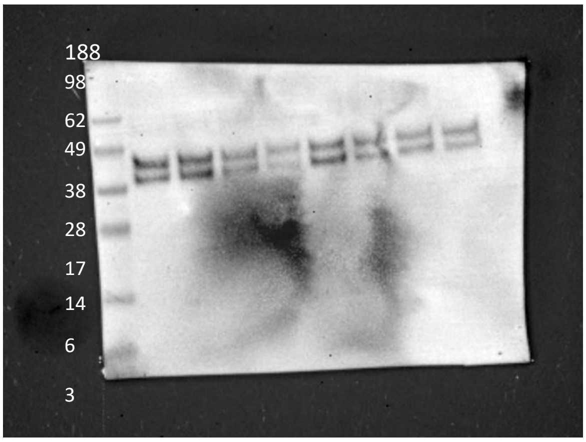

1 CTRL 3 CTRL 4 CTRL 5 CTRL 6 CTRL 2 HnB 4 HnB 6 HnB

GAPDH (~ 36 kDa)

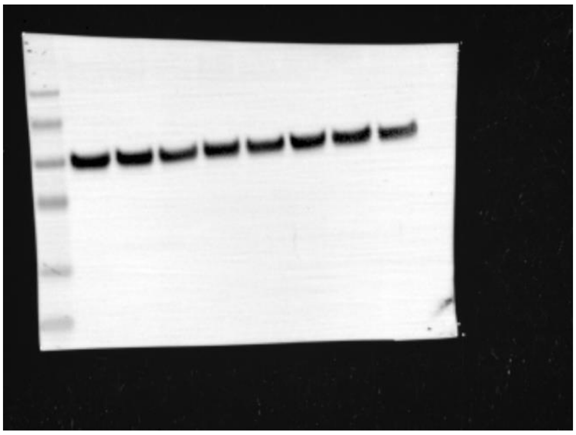

1 CTRL 3 CTRL 4 CTRL 5 CTRL 6 CTRL 2 HnB 4 HnB 6 HnB

GEL C

ERK 1/2 (~ 42/44 kDa)

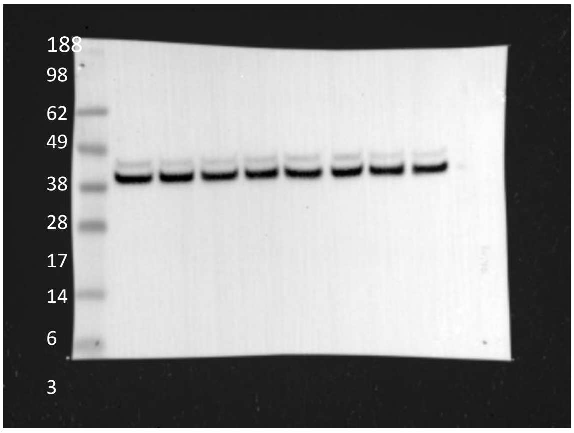

1 CTRL 3 CTRL 4 CTRL 5 CTRL 6 CTRL 2 HnB 4 HnB 6 HnB

GAPDH (~ 36 kDa)

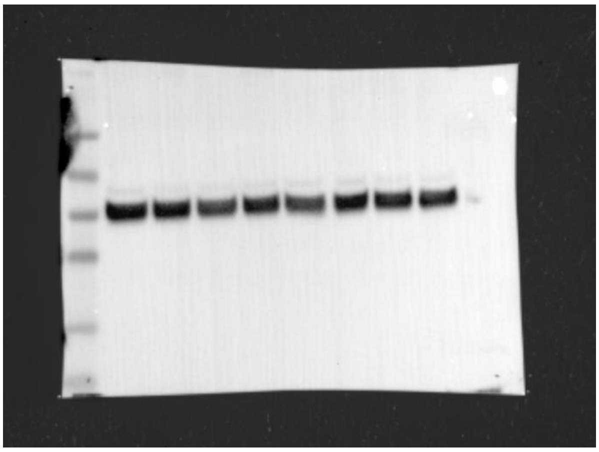

1 CTRL 3 CTRL 4 CTRL 5 CTRL 6 CTRL 2 HnB 4 HnB 6 HnB

Nucleus Accumbens

GEL D

p- ERK 1/2 (~ 42/44 kDa)

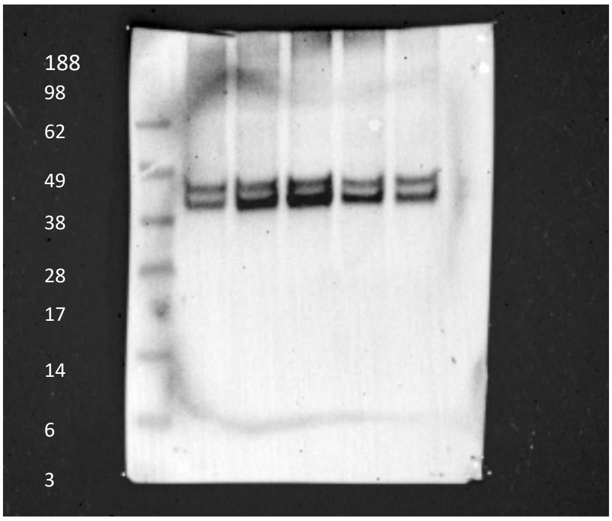

1 CTRL 3 CTRL 1 HnB 3 HnB 5 HnB

GAPDH (~ 36 kDa)

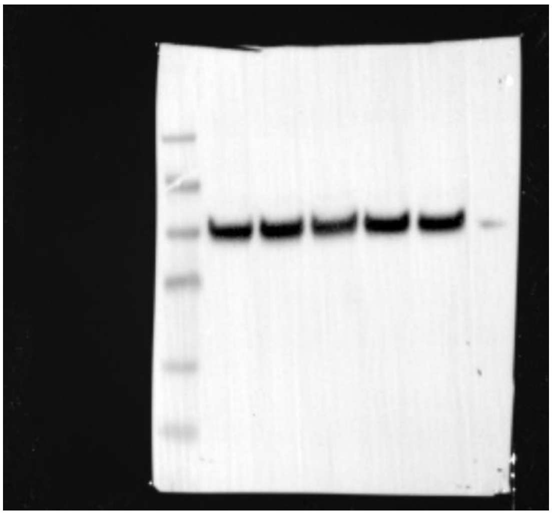

1 CTRL 3 CTRL 1 HnB 3 HnB 5 HnB

GEL D

ERK 1/2 (~ 42/44 kDa)

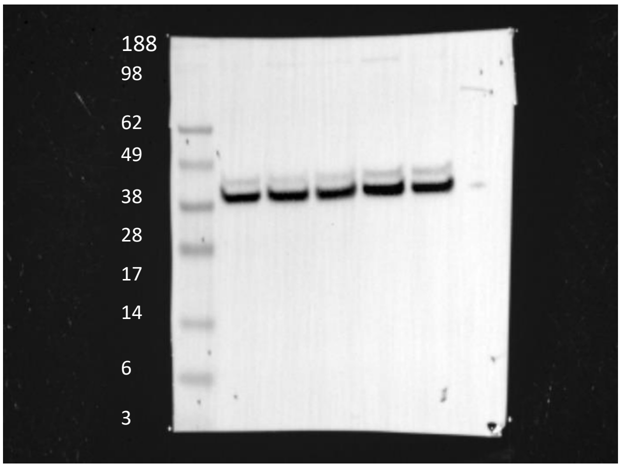

1 CTRL 3 CTRL 1 HnB 3 HnB 5 HnB

GAPDH (~ 36 kDa)

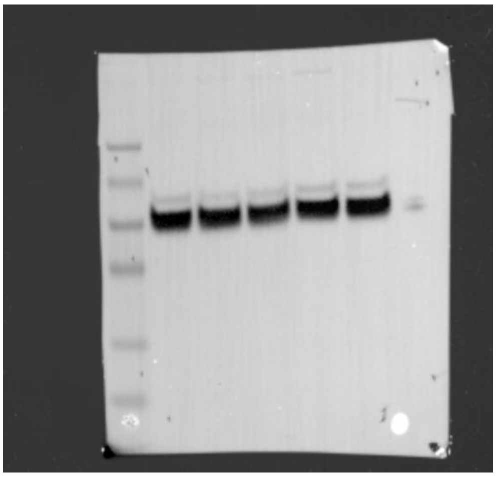

1 CTRL 3 CTRL 1 HnB 3 HnB 5 HnB

WESTERN BLOT IMAGES

Ventral Tegmental Area

GEL A

p- AKT (~ 60 kDa)

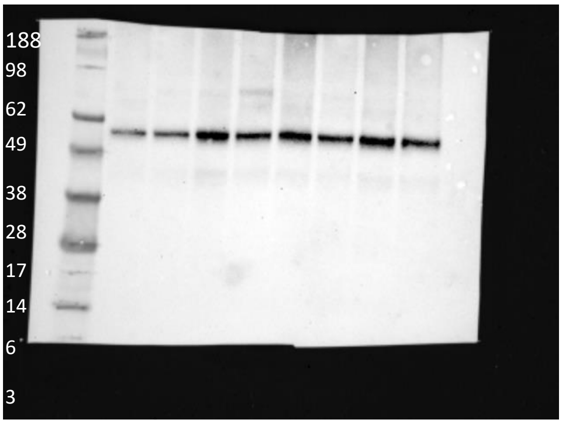

1 CTRL 2 CTRL3 CTRL 4 CTRL 1 HnB 2 HnB 3 HnB 4 HnB

GAPDH (~ 36 kDa)

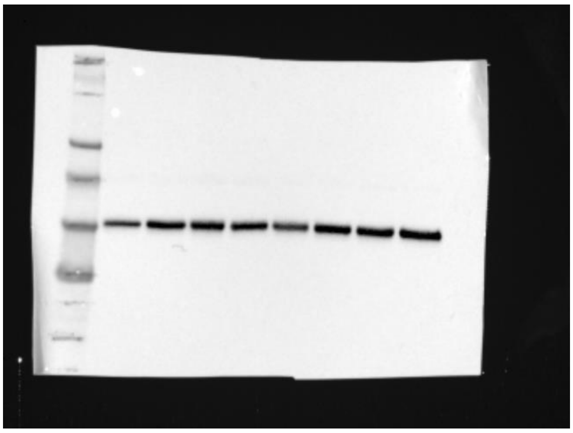

1 CTRL 2 CTRL 3 CTRL 4 CTRL 1 HnB 2 HnB 3 HnB 4 HnB

GEL A

AKT (~ 60 kDa)

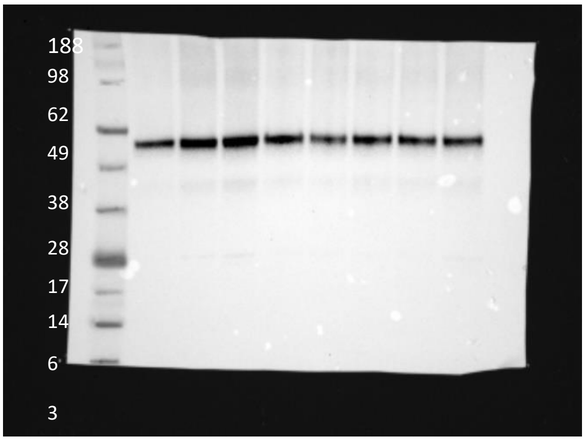

1 CTRL 2 CTRL 3 CTRL 4 CTRL 1 HnB 2 HnB 3 HnB 4 HnB

GAPDH (~ 36 kDa)

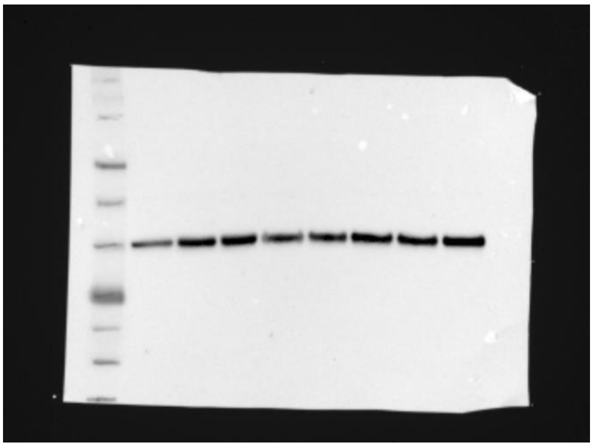

1 CTRL 2 CTRL 3 CTRL 4 CTRL 1 HnB 2 HnB 3 HnB 4 HnB

Ventral Tegmental Area

GEL B

p- AKT (~ 60 kDa)

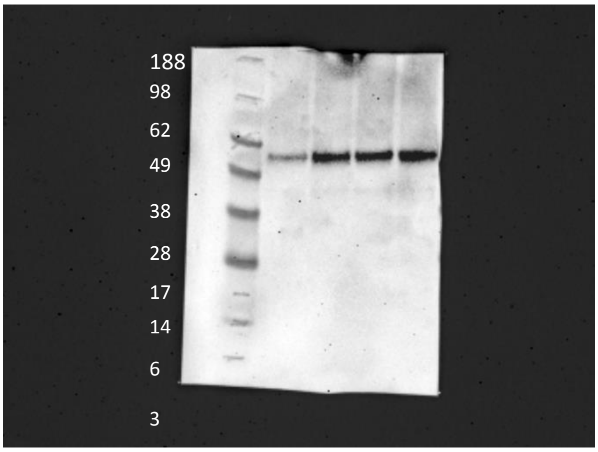

5 CTRL 5 CTRL 6 HnB 6 HnB

GAPDH (~ 36 kDa)

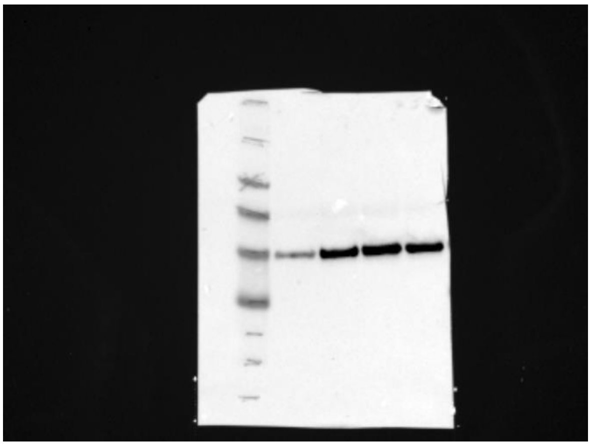

5 CTRL 5 CTRL 6 HnB 6 HnB

GEL B

AKT (~ 60 kDa)

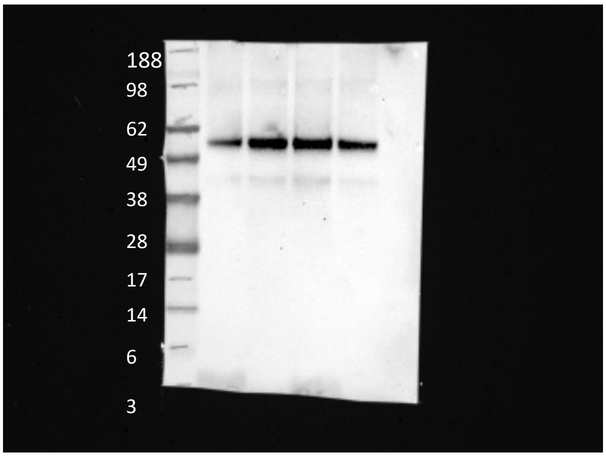

5 CTRL 5 CTRL 6 HnB 6 HnB

GAPDH (~ 36 kDa)

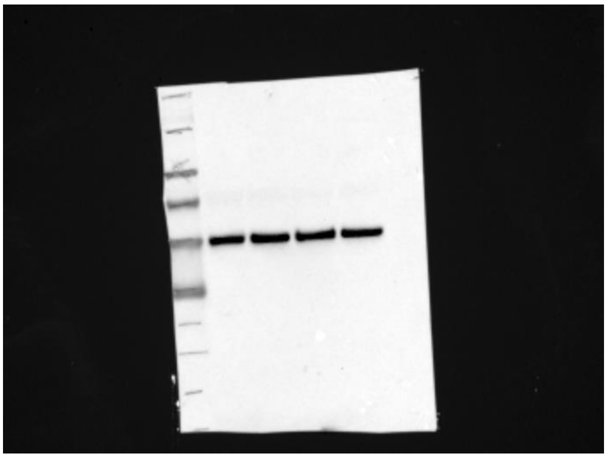

5 CTRL 5 CTRL 6 HnB 6 HnB

Ventral Tegmental Area

GEL C

p- AKT (~ 60 kDa)

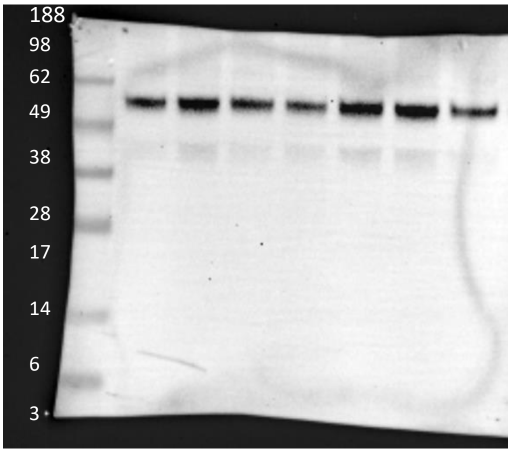

1 CTRL 1 HnB 2 CTRL 2 HnB 3 CTRL 3 HnB 4 CTRL

Representative bands in figure 3a

GAPDH (~ 36 kDa)

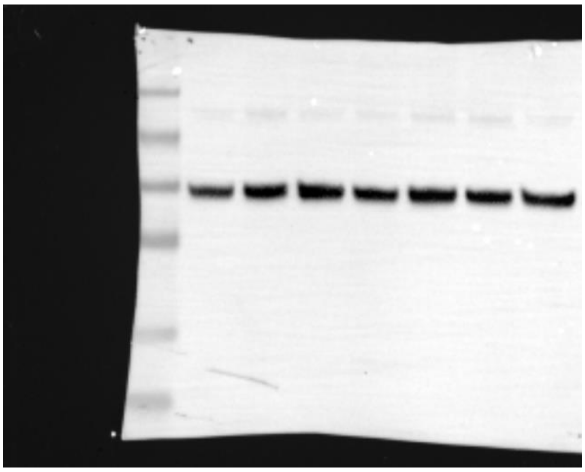

1 CTRL 1 HnB 2 CTRL 2 HnB 3 CTRL 3 HnB 4 CTRL

Representative bands in figure 3a

GEL C

AKT (~ 60 kDa)

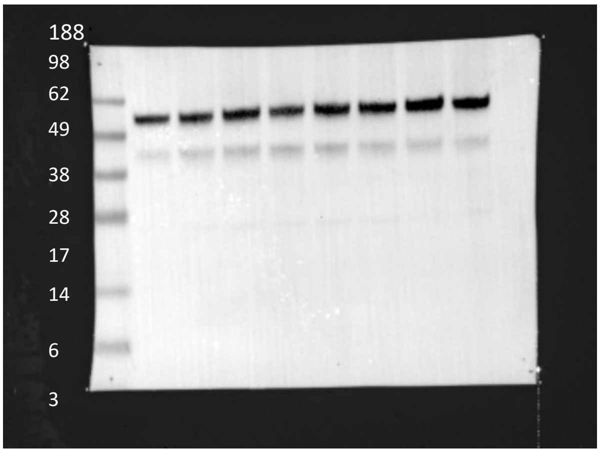

1 CTRL 1 HnB 2 CTRL 2 HnB 3 CTRL 3 HnB 4 CTRL

Representative bands in figure 3a

GAPDH (~ 36 kDa)

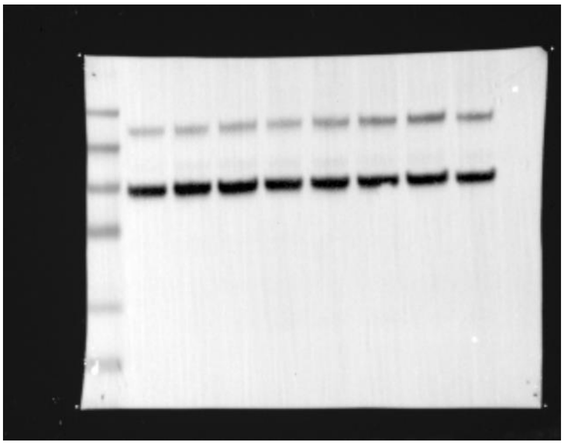

1 CTRL 1 HnB 2 CTRL 2 HnB 3 CTRL 3 HnB 4 CTRL

Representative bands in figure 3a

Ventral Tegmental Area

GEL D

p- AKT (~ 60 kDa) **GAPDH** (~ 36 kDa)

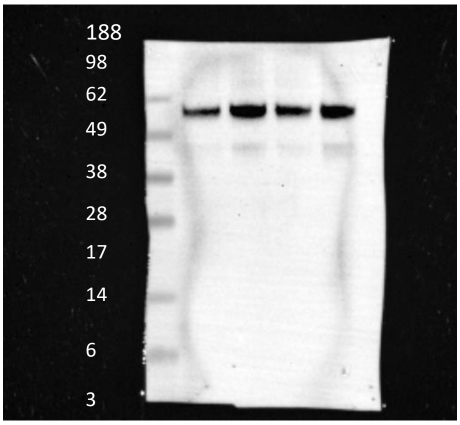

5 CTRL 5 HnB 6 CTRL 6 HnB

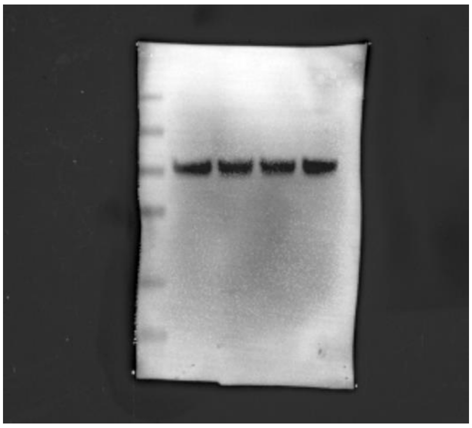

5 CTRL 5 HnB 6 CTRL 6 HnB

GEL D

AKT (~ 60 kDa) **GAPDH** (~ 36 kDa)

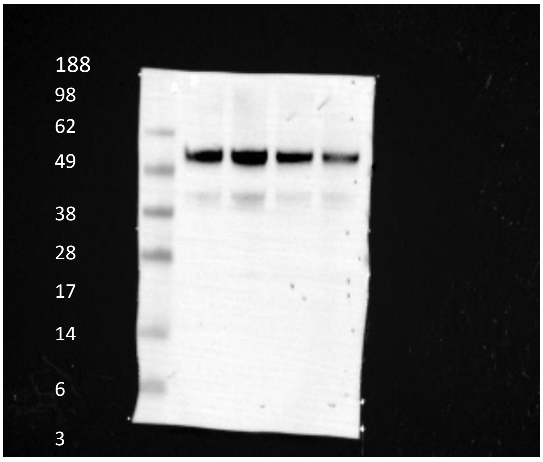

5 CTRL 5 HnB 6 CTRL 6 HnB

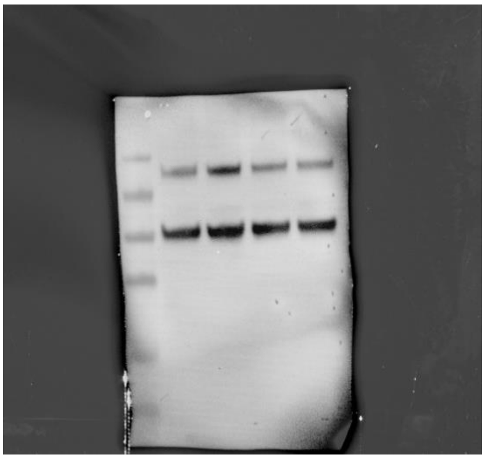

5 CTRL 5 HnB 6 CTRL 6 HnB

Ventral Tegmental Area

GEL A

p- ERK 1/2 (~ 42/44 kDa)

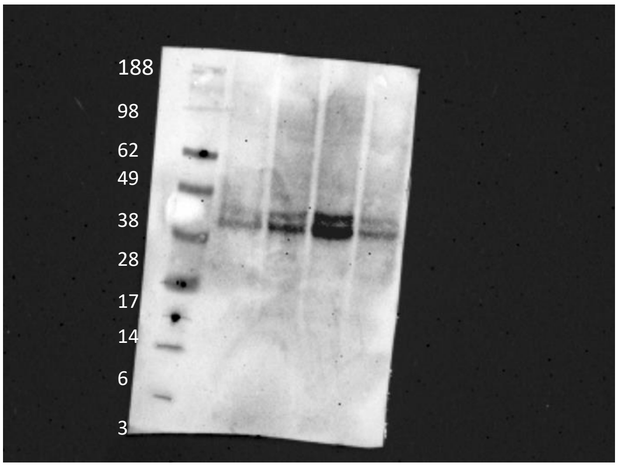

1 CTRL 2 CTRL 1 HnB 2 HnB

GAPDH (~ 36 kDa)

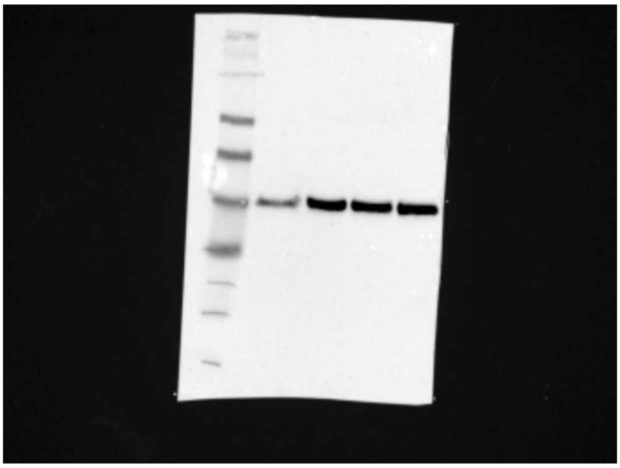

1 CTRL 2 CTRL 1 HnB 2 HnB

GEL A

ERK 1/2 (~ 42/44 kDa)

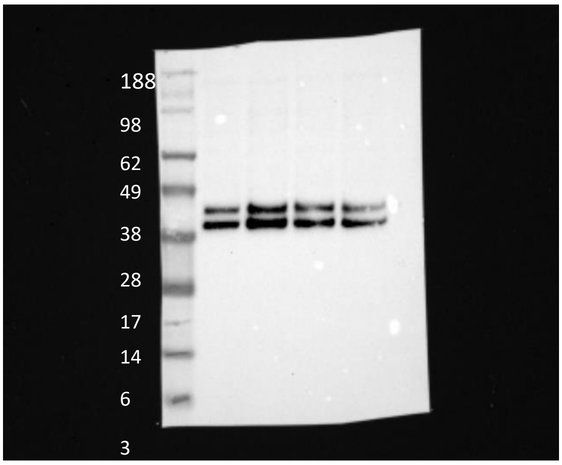

1 CTRL 2 CTRL 1 HnB 2 HnB

GAPDH (~ 36 kDa)

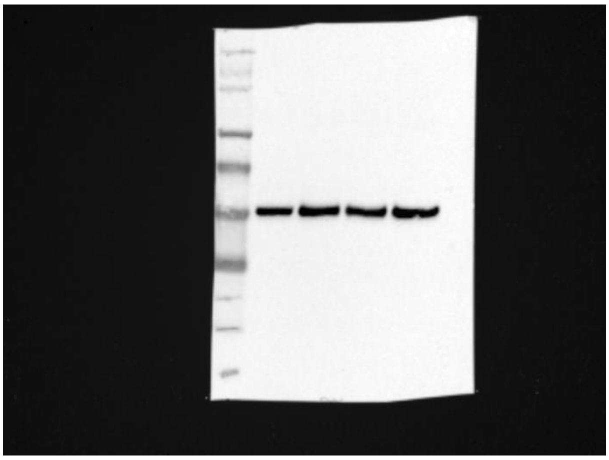

1 CTRL 2 CTRL 1 HnB 2 HnB

Ventral Tegmental Area

GEL B

p- ERK 1/2 (~ 42/44 kDa)

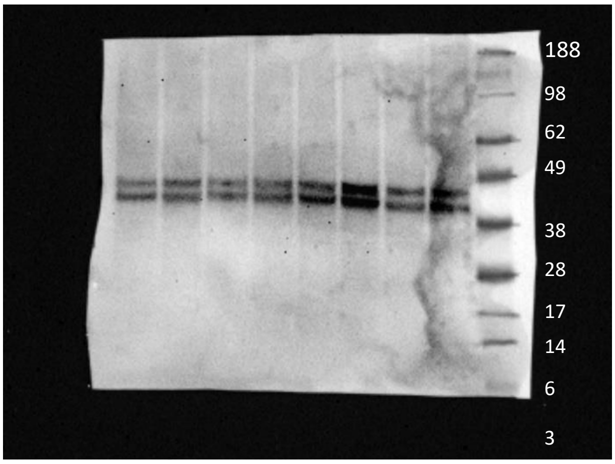

6 HnB 5 HnB 4 HnB 3 HnB 6 CTRL 5 CTRL 4 CTRL 3 CTRL

GAPDH (~ 36 kDa)

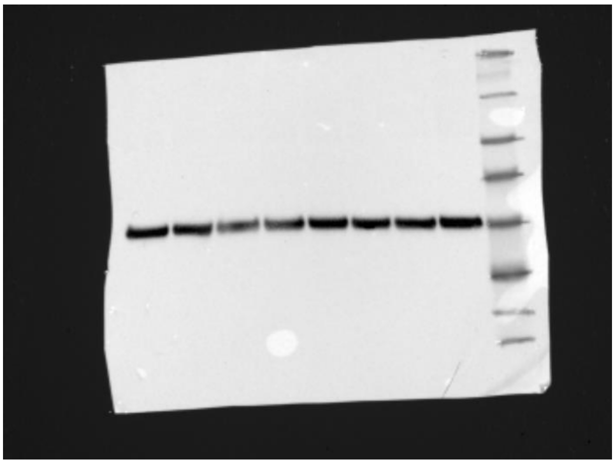

6 HnB 5 HnB 4 HnB 3 HnB 6 CTRL 5 CTRL 4 CTRL 3 CTRL

GEL B

ERK 1/2 (~ 42/44 kDa)

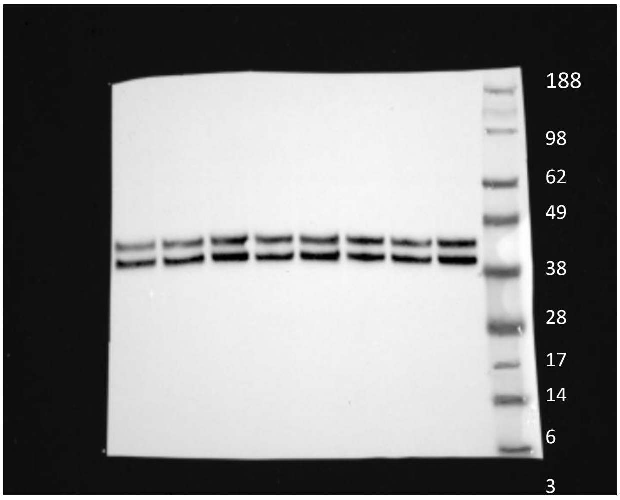

6 HnB 5 HnB 4 HnB 3 HnB 6 CTRL 5 CTRL 4 CTRL 3 CTRL

GAPDH (~ 36 kDa)

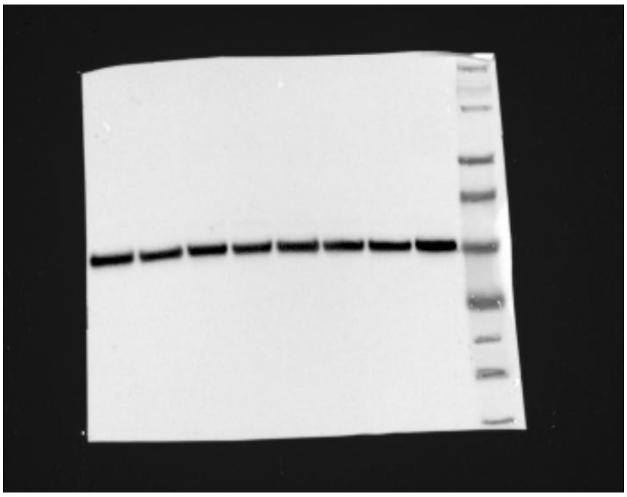

6 HnB 5 HnB 4 HnB 3 HnB 6 CTRL 5 CTRL 4 CTRL 3 CTRL

Ventral Tegmental Area

GEL C

p- ERK 1/2 (~ 42/44 kDa)

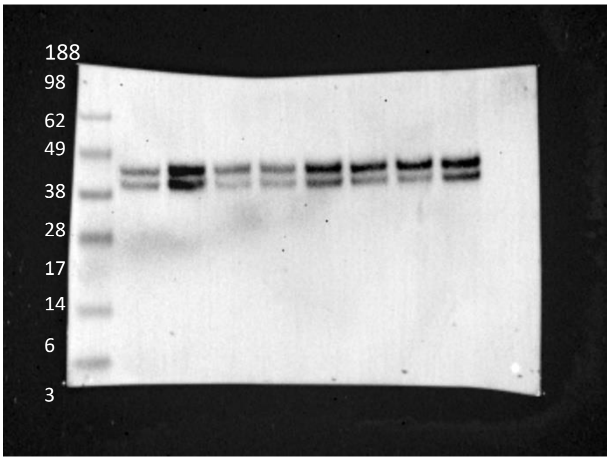

1 CTRL 1 HnB 2 CTRL 2 HnB 3 CTRL 3 HnB 4 CTRL 4 HnB

Representative bands in figure 3b

GAPDH (~ 36 kDa)

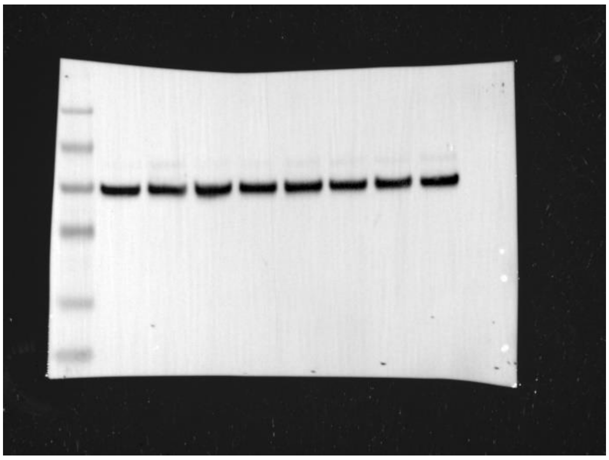

1 CTRL 1 HnB 2 CTRL 2 HnB 3 CTRL 3 HnB 4 CTRL 4 HnB

Representative bands in figure 3b

GEL C

ERK 1/2 (~ 42/44 kDa)

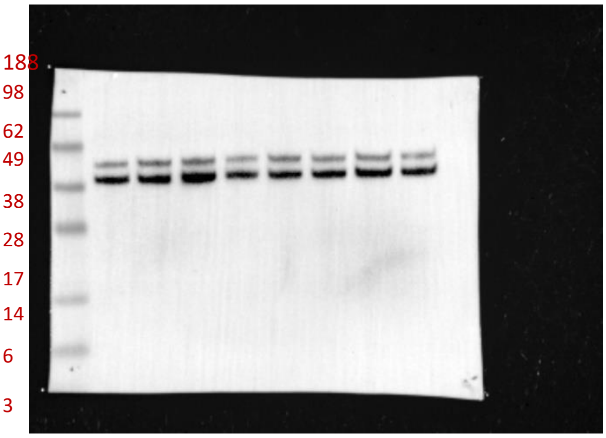

1 CTRL 1 HnB 2 CTRL 2 HnB 3 CTRL 3 HnB 4 CTRL 4 HnB

Representative bands in figure 3b

GAPDH (~ 36 kDa)

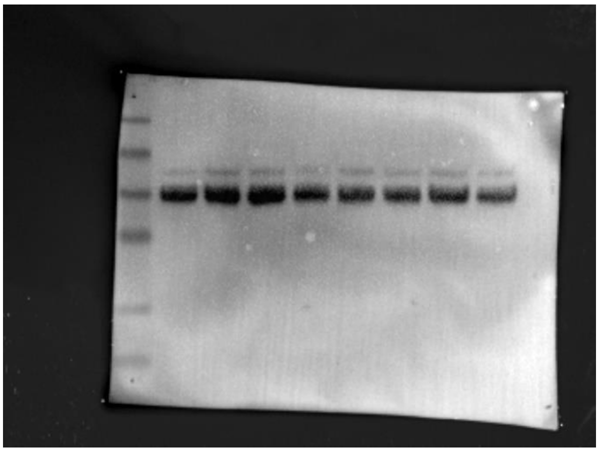

1 CTRL 1 HnB 2 CTRL 2 HnB 3 CTRL 3 HnB 4 CTRL 4 HnB

Representative bands in figure 3b

Ventral Tegmental Area

GEL D

p- ERK 1/2 (~ 42/44 kDa) | **GAPDH** (~ 36 kDa)

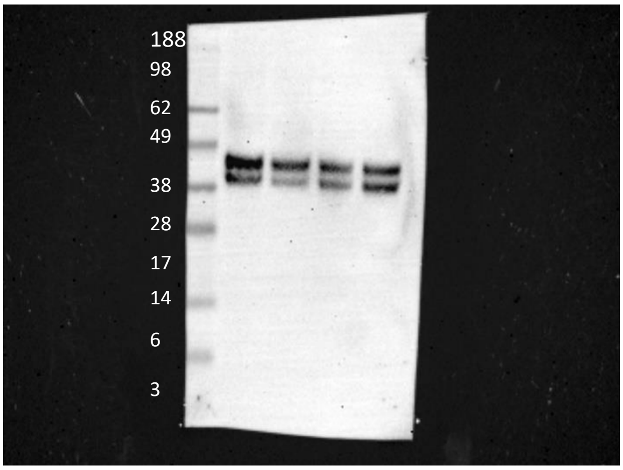

5 CTRL 5 HnB 6 CTRL 6 HnB

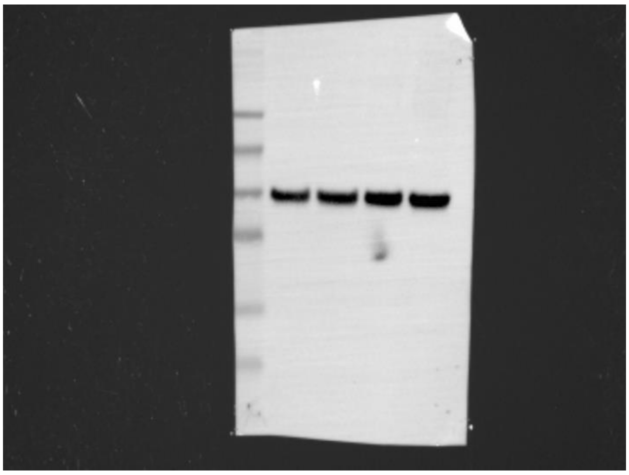

5 CTRL 5 HnB 6 CTRL 6 HnB

GEL D

ERK 1/2 (~ 42/44 kDa) | **GAPDH** (~ 36 kDa)

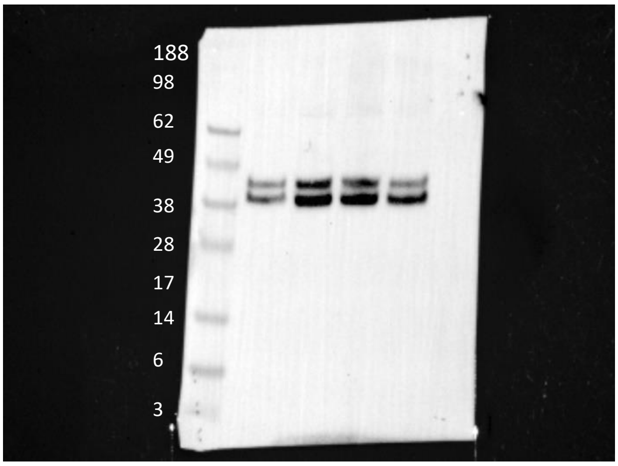

5 CTRL 5 HnB 6 CTRL 6 HnB

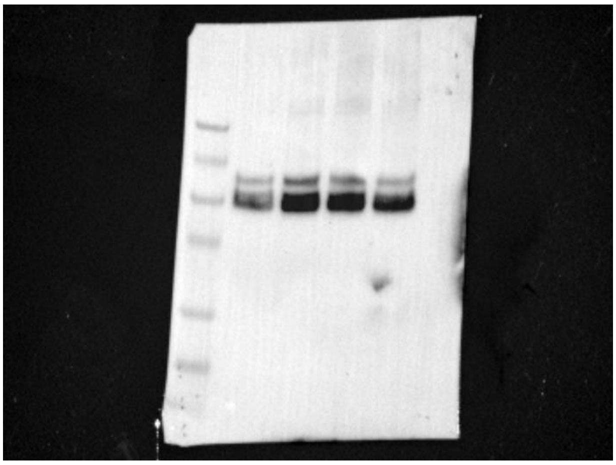

5 CTRL 5 HnB 6 CTRL 6 HnB
